# Supplementary material for: What does it take to learn the rules of RNA base pairing? A lot less than you may think
Source: bioRxiv. 2025 Aug 2:2025.07.31.668042. Preprint. [Version 1] doi: 10.1101/2025.07.31.668042 (PMC12324431; doi:10.1101/2025.07.31.668042)
Supplement: Supplement 1 [file media-1.gz › supplemental_material/src/rscape_v2.6.0.tar.gz!/rscape_v2.6.0/lib/hmmer/documentation/userguide/inclusions/hmmpgmd-search-stats-serialize.pdf]

| | | |
|---|---|---|
| Elapsed (8 bytes) | | |
| User (8 bytes) | | Sys (8 bytes) |
| Z (8 bytes) | | DomZ (8 bytes) |
| Z_setby (1 byte) | DomZ_setby (1 byte) | Nmodels (8 bytes) |
| Nseqs (8 bytes) | | N_past_msv (8 bytes) |
| N_past_bias (8 bytes) | | N_past_vit (8 bytes) |
| N_past_fwd (8 bytes) | | Nhits (8 bytes) |
| Nreported (8 bytes) | | Nincluded (8 bytes) |
| Hit_offsets (nhits * 8 bytes) | | |
